# Spastic Paraplegia Type 30 Associated with Levodopa‐Responsive Parkinsonism

**DOI:** 10.1002/mdc3.13815

**Published:** 2023-07-07

**Authors:** Amy Gallagher, Conor Fearon, Kathryn Smith, Timothy Lynch

**Affiliations:** ^1^ Department of Neurology Dublin Neurological Institute at the Mater Misericordiae University Hospital Dublin Ireland; ^2^ Department of Neurology St Vincent's University Hospital Dublin Ireland; ^3^ Health Affairs, University College Dublin Ireland

**Keywords:** parkinsonism, levodopa, genetic, spasticity, hereditary, paraplegia

More than 80 genes have been linked with hereditary spastic paraparesis (HSP). HSP's clinical presentation can be heterogenous, resulting in a broad range of symptoms in addition to characteristic leg spasticity. Parkinsonism can be a feature of some HSP subtypes, but has never been described in hereditary spastic paraparesis 30 (spastic paraplegia 30 or *SPG30*).^1^

A 51‐year‐old, right‐handed woman presented with progressive gait deterioration. She had no medical history of note and no family history of any neurological conditions nor gait problems (Fig. 1). Her birth and development were normal. Walking was an issue for her whole life, becoming more apparent in her teens and early adulthood and particularly troublesome from age 50 onward. She described a sensation of being “dragged forward” with her knees pulled toward one another. She found it difficult to place her heels on the ground while walking, preferring to walk on her toes. At her first presentation she also reported urinary urgency and nocturia without incontinence. Her arm function, speech, hearing, and vision were unimpaired.

**FIG. 1:**
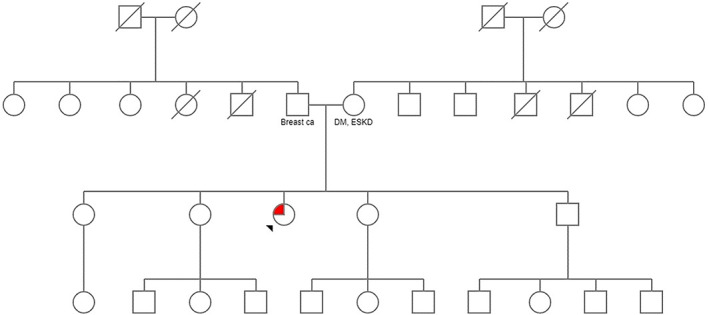
Patient pedigree.

Cranial nerve examination revealed subtle saccadic vertical pursuits and was otherwise normal. She had pathologically brisk reflexes with a spastic catch in her limbs, and Hoffman's sign was negative. There was a marked spastic catch in both legs with loss of muscle bulk, proximal 4/5 leg weakness, and upgoing plantars bilaterally. There was sustained ankle clonus on the right more than left. She had a brisk deep abdominal reflex. Sensation was normal. She walked on her toes with a scissoring gait. Magnetic resonance imaging (MRI) brain showed mild superior frontoparietal atrophy and MRI spine was normal. Next generation sequencing panel revealed a heterozygous missense mutation located on exon 6 of KIF1A: c.[499C > T];[=], p.[(Arg167Cys)];[(=)], consistent with a diagnosis of *SPG30*.

Re‐examination at age 53 revealed spasticity and asymmetric parkinsonism, with hypomimia, bradykinesia, and rigidity more on the left than right, retropulsion leading to falls and depression (Beck's Depression Inventory score 34/63). There was no tremor. A subsequent dopamine transporter scan (DaTscan) was abnormal, revealing markedly reduced striatal radioligand uptake more pronounced on the right, consistent with neurodegenerative parkinsonism (Fig. 2). Her bradykinesia, rigidity, and frequency of falls improved with levodopa/carbidopa up to a dose of 250/1000 mg/day with an improvement in the Movement Disorder Society‐Unified Parkinson's Disease Rating Scale part III to 40 (*on*) from 55 (*off*). The patient developed wearing off after 2 h, freezing of gait and cognitive impairment with a Montreal Cognitive Assessment of 16/30 (with deficits in attention, language, abstraction, visuospatial, and executive function) on review at age 58 (video 1). She reported a period of well‐formed visual hallucinations 3 years after developing parkinsonism, however, these subsequently settled without treatment, and she did not experience any extracampine hallucinations. Other non‐motor symptoms included depression, anxiety, fatigue, urinary frequency, and constipation.

**FIG. 2:**
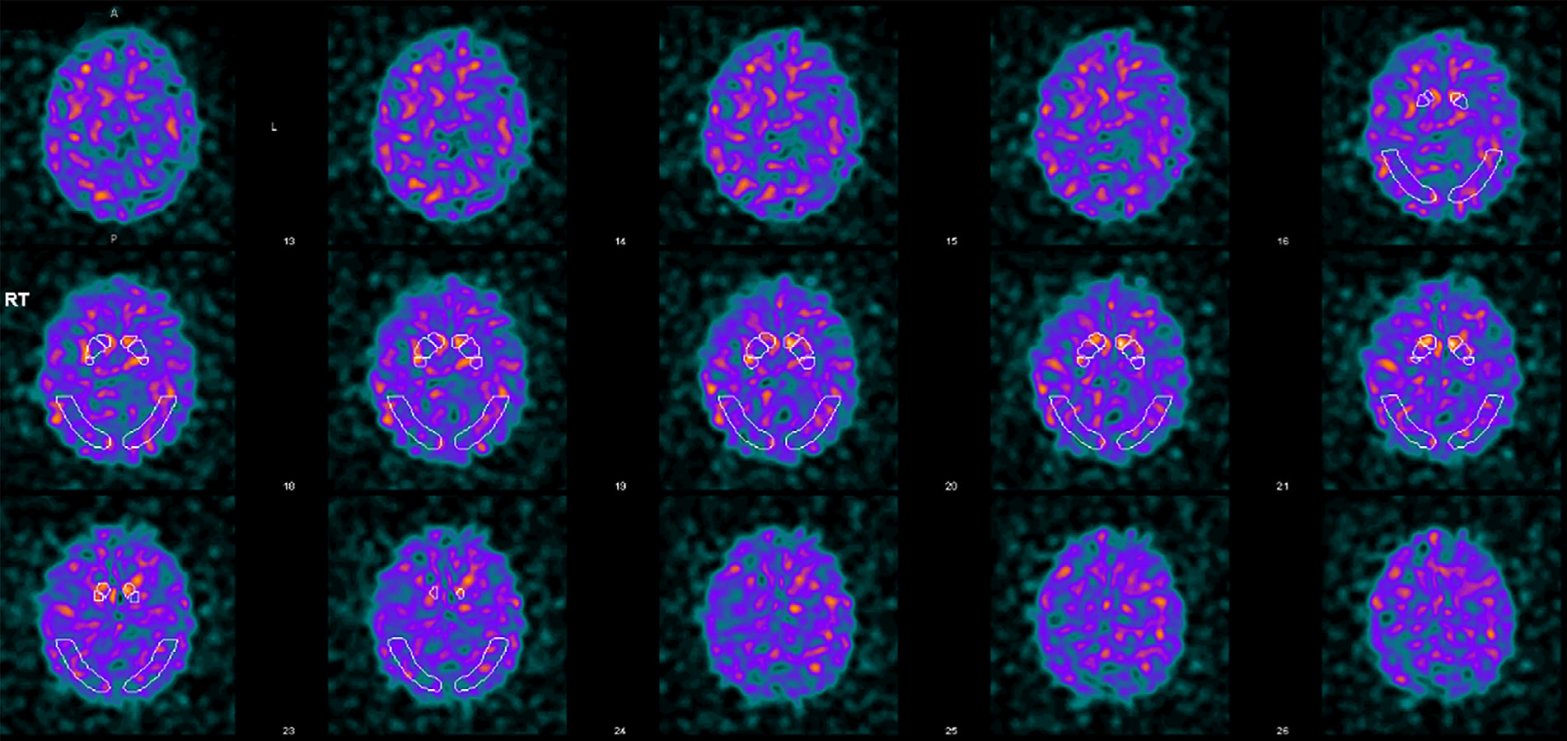
Dopamine transporter scan 3 years after developing parkinsonism.

**Video 1:** Clinical examination 4 years after developing parkinsonism, demonstrating hypomimia, hypophonia, saccades and eyelid opening, spasticity, and asymmetric bradykinesia. This examination was completed 5 h after levodopa therapy in the “*off*” state.

HSP is a group of autosomal genetic disorders predominantly affecting the legs causing spasticity and weakness. More than 80 subtypes of HSP have been defined to date, each associated with a different genetic locus. *SPG30* is caused by pathogenic variants in KIF1A and accounts for just 5% of autosomal dominant HSP cases.^1^ To date there are no published neuropathological studies of *SPG30*.

Parkinsonism has previously been described in association with SPG7, SPG11, SPG15, SPG21, SPG35, SPG48, and SPG78,^1^, ^2^, ^3^, ^4^ but not with SPG30. Kara et al^5^ assessed 30 cases of SPG11, five of whom had parkinsonism, and three of these five reported benefit associated with levodopa. Another 2018 study of 22 patients with SPG11 revealed parkinsonism in six (27.3%) of mainly rigid‐akinetic type, similar to our patient's presentation, with rest tremor present in only three patients. This study, however, showed no significant improvement in UPDRS‐III score with levodopa therapy. DaTscan imaging confirmed significantly lower uptake in the striatum bilaterally compared with controls, irrespective of whether there was clinically evident parkinsonism, reflecting the possibility that striatal degeneration could be a universal process in SPG11.^6^ A case report published by Paisán‐Ruiz et al^7^ describes one further case of SPG11 with levodopa‐responsive parkinsonism and bilateral decreased striatal uptake on DaTscan. The patient also developed visual hallucinations, although this was in the context of ropinirole therapy.

Levodopa benefit has been recorded in SPG7 and in all six SPG15 patients with parkinsonism described by Milagres Araujo et al.^3^, ^8^ Four of these SPG15 patients had single‐photon emission computed tomography imaging with three showing symmetrical nigrostriatal degeneration and one with asymmetric nigro‐striatal loss.

This is the first report of levodopa‐responsive parkinsonism in a patient with SPG30. A 2020 literature review of 86 SPG30 cases did not reveal parkinsonism as a feature in any reported cases.^9^ It is possible that the patient developed idiopathic Parkinson's disease at age 53 that caused decompensation of her longstanding SPG30, given the levodopa response, motor fluctuations, visual hallucinations, and rapid progression after parkinsonism.

Kinesin‐like protein KIF1A is a microtubule motor protein, or kinesin, involved in the antegrade transport of vesicles required for pre‐ and post‐synaptic assembly, autophagy, and neuron survival. These vesicles transport membrane proteins such as vesicular monoamine transporter‐2 (VMAT), which in turn acts to concentrate monoamines into presynaptic vesicles. VMAT‐2 is also concentrated in nigrostriatal dopaminergic neurons; of note increased nigrostriatal degeneration was observed in VMAT‐2 knockout mouse models.^10^

We describe a patient with levodopa‐responsive parkinsonism and genetically confirmed SPG30. Both levodopa‐responsive and nonresponsive cases of parkinsonism have been described in other hereditary spastic parapareses. We report here the first patient with a *KIF1A* mutation resulting in SPG30 also with levodopa‐responsive parkinsonism, possibly indicating that this novel presentation results from nigrostriatal degeneration secondary to VMAT‐2 dysfunction.

## Author Roles

(1) Patient Care. (2) Manuscript preparation: A. Writing of the First Draft, B. Review and Critique.

A.G.: 1, 2A.

C.F.: 2B.

K.S.: 2A.

T.L.: 1, 2B.

## Disclosures

**Ethical Compliance Statement:** The authors confirm that the approval of an institutional review board was not required for this work. The patient provided written informed consent for the purposes of this publication. We confirm that we have read the journal's position on issues involved in ethical publication and affirm that this work is consistent with those guidelines.

**Funding Sources and Conflicts of Interest:** No specific funding was received for this work and the authors declare that there are no conflicts of interest relevant to this work.

**Financial Disclosures for the Previous 12 Months:** T.L.'s research is supported by The Michael J. Fox Foundation, the Health Research Board Ireland, EU JPND/HRB, and the Irish Institute of Clinical Neuroscience. C.F.'s research is supported by The Michael J. Fox Foundation. A.G.'s research is supported by the Health Research Board Ireland.
